# As_2_S_2_ Mediates the ROS/P38 MAPK Signaling Pathway to Induce Apoptosis and S-Phase Arrest in Myelodysplastic Syndrome Cells

**DOI:** 10.3390/cimb47040253

**Published:** 2025-04-07

**Authors:** Pengjie Chen, Li Yu, Rui Yang, Wen Zeng, Yanxi Chen, Fengmei Wang, Yonggang Xu, Xiupeng Yang

**Affiliations:** Department of Hematology, Xiyuan Hospital, China Academy of Chinese Medical Sciences, Beijing 100091, China

**Keywords:** ROS, P38 MAPK, As_2_S_2_, myelodysplastic syndrome, apoptosis

## Abstract

Myelodysplastic syndrome (MDS) is a heterogeneous myeloid clonal disorder that represents a significant threat to human health. As_2_S_2_, a natural compound, has been shown to exert therapeutic effects on various malignant tumors, including acute myeloid leukemia (AML), breast cancer, and osteosarcoma, based on extensive clinical experience. In this study, we investigated the mechanism by which As_2_S_2_ inhibits the proliferation of the myelodysplastic syndrome (MDS) SKM-1 cell line. Our findings revealed that As_2_S_2_ inhibited the proliferation of SKM-1 cells in a time- and dose-dependent manner. Flow cytometry, protein immunoblotting, and real-time fluorescence quantitative PCR analyses demonstrated that As_2_S_2_ promotes the phosphorylation of P38 MAPK, thereby activating the MAPK signaling pathway. Additionally, it promotes apoptosis by increasing the BAX/Bcl-2 ratio and induces S-phase arrest through the downregulation of the cell cycle-related protein cyclin A2. Further studies demonstrated that As_2_S_2_-treated cells exhibited ROS accumulation under fluorescence microscopy, along with activation of the P38 MAPK signaling pathway, increased apoptosis, and S-phase arrest in the cell cycle. This process could be partially reversed by the ROS inhibitor N-acetylcysteine. Therefore, the results of the present study suggest that As_2_S_2_ induces ROS accumulation in SKM-1 cells, which contributes to the activation of the P38 MAPK signaling pathway, promoting apoptosis and S-phase arrest in the cell cycle. Additionally, As_2_S_2_ may serve as a potent therapeutic agent for the treatment of myelodysplastic syndromes, with ROS acting as one of the key therapeutic targets.

## 1. Introduction

Myelodysplastic syndrome (MDS) is a myeloid hematological malignancy characterized by the clonal proliferation of hematopoietic stem cells, myeloid dysplasia, peripheral hematopoiesis, ineffective hematopoiesis, and the risk of progression to acute myeloid leukemia (AML) [1,2]. As a disease primarily affecting the elderly, the median age at diagnosis is 71–76 years, with only 6% of cases diagnosed in individuals aged ≤ 50 years [3]. This presents a significant threat to the health of the aging population. Currently, the primary treatment strategy for MDS focuses on symptom management, typically using erythropoiesis-stimulating agents or demethylating drugs such as azacitidine and decitabine. Hematopoietic stem cell transplantation remains the only potential cure [4], but it is not suitable for the majority of patients due to factors such as advanced age, donor compatibility, and the high cost of treatment. The search for effective and cost-effective drugs derived from natural substances is one of the prominent areas of current research. As_2_S_2_ is a natural drug that has been used for many years, with demonstrated efficacy in the treatment of hematologic malignancies, including acute myeloid leukemia (AML) [5], diffuse large B-cell lymphoma [6], and myelodysplastic syndrome (MDS) [7], as well as other cancers such as osteosarcoma [8] and breast cancer [9]. The effect of As_2_S_2_ on malignant tumors involves a complex biological process, influencing several biological mechanisms, such as DNA demethylation, mitochondrial autophagy, and the release of cytokines and reactive oxygen species (ROS) [10,11]. This, in turn, induces cell cycle arrest and apoptosis, which are among the mechanisms through which As_2_S_2_ exerts its therapeutic effects in the treatment of malignant tumors.

Reactive oxygen species (ROS) are by-products of mitochondrial metabolism that regulate the proliferative and differentiation capacity of cells by affecting signal transductions and redox reactions [12]. High levels of ROS generated in response to drug or stress stimuli inhibit cell proliferation and may induce cell death, a process involving the MAPK signaling pathway [13,14]. The MAPK signaling pathway, which transmits extracellular signals to the nucleus through cascading phosphorylation reactions, holds significant potential in cancer therapy. Jian Liu et al. [15] found that jaceosidin (JAC) induced apoptosis and G0/G1 phase arrest in gastric cancer cells by accumulating ROS, which activates the P38 MAPK signaling pathway. Rabdoternin E can induce massive ROS accumulation and trigger apoptosis and ferroptosis in lung cancer cells through the activation of the ROS/P38 MAPK/JNK signaling pathway [16]. These natural compounds elevate ROS levels to activate the MAPK signaling pathway, leading to cell cycle arrest and apoptosis in tumor cells.

## 2. Materials and Methods

### 2.1. Key Materials and Equipment

RPMI medium 1640 (Gibco, Suzhou, China, Lot: 6124222); fetal bovine serum (Cegrogen, Stadtallendorf, Germany, Lot: A0500-3010/P211102); As_2_S_2_ was obtained from the pharmacy of Xiyuan Hospital, China Academy of Traditional Chinese Medicine(Beijing, China); SB203580 (MCE, Monmouth Junction, NJ, USA, Lot: 318906); N-acetylcysteine (MCE, Monmouth Junction, NJ, USA, Lot: 243751); RIPA buffer (Solarbio, Beijing, China, Lot: 240003004); BCA protein assay kit (Beyotime, Shanghai, China, P0012); one-step PAGE gel preparation kit (Affinibody, Wuhan, China, Lot: W2401B810X); TRIzol (Thermo Fisher Scientific, Waltham, MA, USA, Lot: 99090201); fasting one-step de-genomic cDNA first strand synthesis premixed reagent (Tiangen, Beijing, China, Lot: A0417A); PowerUp™ SYBR™ Green (Applied Biosystems, Foster City, CA, USA, Lot: 3001959); annexin V-FITC/PI apoptosis kit (Elabscience, Wuhan, China, Lot: AK21271); DNA content quantitation assay (Solarbio, Suzhou, China, Lot: 20231011); reactive oxygen species assay kit (Beyotime, Shanghai, China, Lot: Z905241031); cell counting kit-8 (Solarbio, Suzhou, China, Lot: 240010008); omni-ECL (Epyzyme, Shanghai, China, Lot: 038A3300); tricolor prestained protein marker (Epyzyme, Shanghai, China, Lot: 028811600); p38 MAPK, cyclin A2 (Biodragon, Suzhou, China, Lot: W1227P, B28RC111); BAX/Bcl-2 (Servicebio, Wuhan, China, Lot: GB114122/GB113375); phospho-P38 MAPK/HRP-conjugated affinipure goat anti-mouse IgG (H + L) (Proteintech, Wuhan, China, Lot: 00161916, 20001101).

Fluorescence quantitative PCR instrument (9600+) (BIOER, FQD-96 A, Shanghai, China); UV–visible spectrophotometer (NanoReady, FC-1100, Beijing, China); protein blotting electrophoresis system (BIO-RAD, PowerPac™# 1645050, Hercules, CA, USA); iBright™ imaging system for fluorescence colorimetric analysis (Thermo, iBright1500, Waltham, MA, USA); flow cytometer (Beckman Coulter, NAVIOS, Brea, CA, USA); fluorescence imaging system (ZEISS, Axio Imager M2, Dublin, CA, USA).

### 2.2. Cell Culture

The SKM-1 cell line was generously provided by the First Affiliated Hospital of Soochow University and cultured in RPMI-1640 medium containing 10% FBS, 100 U/mL penicillin, and 100 μg/mL streptomycin at 37 °C with 5% CO_2_.

### 2.3. CCK-8 Cell Viability Assay

Cells were cultured in 96-well plates at a density of 3 × 10^4^ cells/well. After 12 h of acclimatization, the cells were treated with 0, 1, 2, 4, 8, and 16 μM As_2_S_2_. After 12, 24, and 48 h of incubation, 10 μL of CCK-8 solution was added to each well, and the cells were incubated for an additional 2 h at 37 °C. Absorbance at 562 nm was measured using a microplate reader. All CCK-8 assays were performed in triplicate. The proliferation inhibition rate of the SKM-1 cell line was calculated using the following formula: [(Ac − As)/(Ac − Ab)] × 100%, where As represents the absorbance of the experimental wells, Ac represents the absorbance of the control wells, and Ab represents the absorbance of the blank wells. IC_50_ was calculated using GraphPad Prism 10.2.3 statistical software using nonlinear regression analysis. The IC_50_ (4 μM) at the 48 h intervention time was used as the drug concentration to observe the inhibitory effect of As_2_S_2_ on SKM-1 cells in the presence of the P38 MAPK inhibitor or ROS inhibitor, using the method described above.

### 2.4. Flow Cytometric Detection of Apoptosis Levels

After drug treatment, SKM-1 cells were washed with PBS. A total of 3 × 10^5^ cells were collected and resuspended in 500 μL of diluted 1× annexin V binding buffer. Then, 5 μL of annexin-FITC reagent and 5 μL of PI reagent (50 μg/mL) were added to the cell suspension, mixed thoroughly, and incubated in the dark for 15 min. The proportion of apoptotic cells in each group was analyzed using CellQuest Pro software (v5.2, BD Biosciences, Franklin Lakes, NJ, USA). Statistical comparisons between groups were performed using a one-way ANOVA with an LSD post hoc test (Bonferroni-adjusted *p* < 0.0167 for three comparisons). If data violated parametric assumptions, a non-parametric Kruskal–Wallis test with Dunn’s correction was applied.

### 2.5. Detection of Cell Cycle Distribution by Flow Cytometry

After drug treatment, 1 mL of cell suspension (1 × 10^6^ cells/mL) were collected, washed with PBS, and then 500 μL of 70% pre-cooled ethanol was added to fix the cells overnight. The cells were then washed with PBS and resuspended in 100 μL of RNase-A solution. After incubation in a 37 °C water bath for 30 min, 400 μL of PI staining solution was added, and the cells were incubated at 4 °C in the dark for 30 min. The excitation wavelength was recorded using a flow cytometer. Red fluorescence at 488 nm was detected, and cell cycle distribution was analyzed using CellQuest software (version 5.1).

### 2.6. Detection of ROS Accumulation Levels by Flow Cytometry and Immunofluorescence

After drug treatment, cells were adjusted to 5 × 10^6^ cells/mL and incubated with serum-free medium containing a 10 μM/L DCFH-DA probe at 37 °C for 20 min to load the probe. After the cells were thoroughly washed with serum-free medium, the fluorescence intensity of the ROS was recorded using a flow cytometer with an excitation wavelength of 488 nm and analyzed using FlowJo 10.9.0. The washed cells were then used to prepare monolayer cell smears using a cell smear centrifuge, and fluorescence changes were observed using a fluorescence microscope with an optional FITC filter.

### 2.7. Protein Immunoblotting for Protein Expression Levels

After drug treatment, cells were thoroughly washed with PBS buffer and lysed on ice for 30 min using protein extraction lysis buffer. The supernatant was collected after centrifugation at 4 °C and 12,000 rpm for 15 min. Protein concentrations were determined and quantified using the BCA protein assay kit. A total of 24 μg of protein was subjected to sodium dodecyl sulfate-polyacrylamide gel electrophoresis (SDS-PAGE). After electrophoresis, the membrane was blocked, washed, and incubated overnight at 4 °C with the primary antibodies (P38 MAPK, P-P38 MAPK, BAX, BCL-2, cyclin A2, GAPDH). After sufficient washing, the horseradish peroxidase-conjugated goat anti-rabbit IgG (H + L) secondary antibody was added and incubated at room temperature for 1.5 h. Fluorescence detection was performed using the iBright™ imaging system.

### 2.8. q-PCR to Detect mRNA Transcription Levels

Total RNA was extracted from the cells after drug treatment, and the expression levels of target genes and internal reference genes were evaluated by q-PCR. The primer sequences of the target genes are shown in Table 1. The relative expression levels of the target genes and internal reference genes were determined using the amplification curves. The Ct value of each gene was calculated from the amplification curve, and the relative expression of target genes and internal reference genes was calculated using the ΔΔCt method. Statistical differences between groups were analyzed using GraphPad Prism 10.2.3.

### 2.9. Statistical Analysis Methods

Data are presented as the means ± standard deviation ($\bar{X}$ ± SD) from three independent biological replicates. Each experiment was repeated three times, and data from all replicates were pooled for analysis. The normality of data distribution was assessed using the Shapiro–Wilk test (*p* > 0.05), and the homogeneity of variances was confirmed by Levene’s test (*p* > 0.05). Parametric tests (Student’s *t*-test for two groups; one-way ANOVA with LSD post hoc test and Bonferroni adjustment for multiple comparisons) were applied when assumptions were met. If data violated parametric assumptions, non-parametric alternatives (Mann–Whitney U test or Kruskal–Wallis test with Dunn’s correction) were used. Statistical analyses were performed using GraphPad Prism 10.2.3. Dose–response curves (IC_50_) were generated via nonlinear regression analysis, and flow cytometry data were processed using built-in modules for apoptosis quantification. A *p*-value < 0.05 was considered statistically significant.

## 3. Results

### 3.1. As_2_S_2_ Inhibits SKM-1 Cell Proliferation

**Figure 1:**
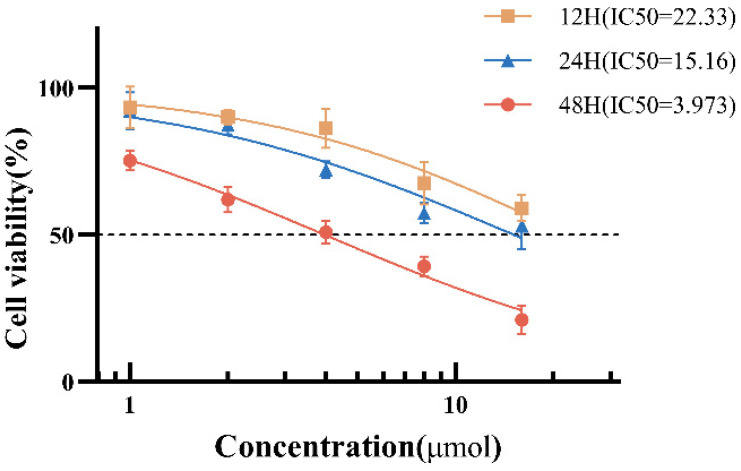
Inhibitory effect of As_2_S_2_ on SKM-1 cells. The inhibitory effect of As_2_S_2_ on SKM-1 cells was time- and dose-dependent, with IC_50_ values of 22.33, 15.16, and 3.973 μM/L at 12, 24, and 48 h, respectively ($\bar{X}$ ± s; *n* = 3).

To investigate the inhibitory effect of As_2_S_2_ on myelodysplastic syndromes, cell viability of SKM-1 cells was assessed using the CCK-8 kit after treatment with 0, 1, 2, 4, 8, and 16 μM As_2_S_2_ for 12, 24, and 48 h, respectively. The cell viability of SKM-1 cells was significantly reduced following drug treatment (Figure 1), with IC_50_ values of 22.33 μM (12 h), 15.61 μM (24 h), and 3.97 μM (48 h), respectively, indicating that As_2_S_2_ inhibited SKM-1 cell proliferation in a time- and dose-dependent manner. To balance therapeutic relevance and mechanistic clarity, we selected 4 μM (near IC_50_) for subsequent experiments as higher concentrations (>8 μM) induced rapid necrosis (>60% cell death within 24 h) which could mask specific apoptotic and cell cycle regulatory effects.

### 3.2. As_2_S_2_ Induces S-Phase Arrest and Apoptosis in the SKM-1 Cell Cycle

**Figure 2:**
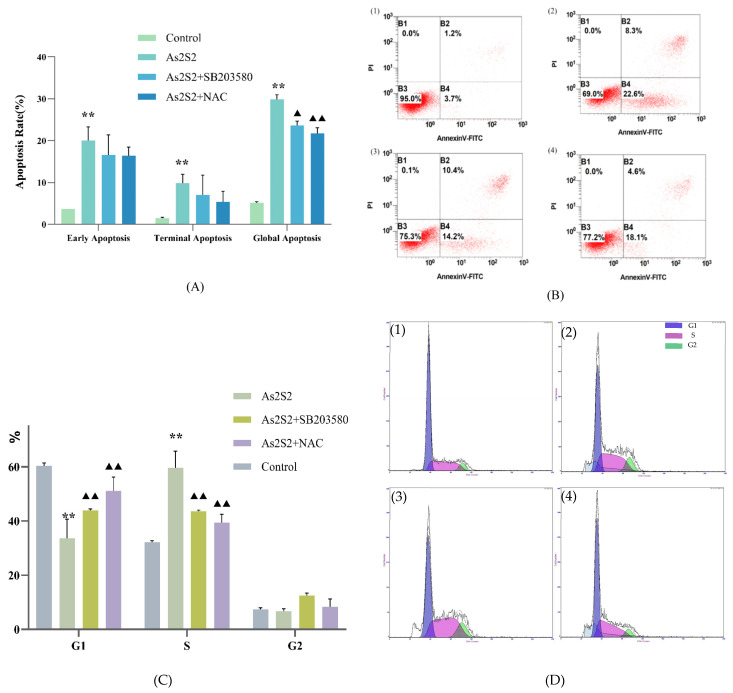
Effects of co-culture of As_2_S_2_ and inhibitors SB203580 (10 μM/L) and NAC (300 mM/L) on apoptosis and cell cycle of SKM-1 cells. (**A**) Effect of co-culture of As_2_S_2_ and the inhibitor SB203580 on apoptosis levels in SKM-1 cells. (**B**) Flow cytometric fluorescence scatter plots of SKM-1 cell apoptosis ((**B(1**)): control group; (**B**(**2**)): As_2_S_2_ group; (**B**(**3**)): As_2_S_2_ and SB203580 co-culture group; (**B**(**4**)): As_2_S_2_ and NAC co-culture group). (**C**) Effect of co-culture of As_2_S_2_ and inhibitors SB203580 and NAC on the cell cycle. (**D**) Histograms of flow cytometry cell cycle distribution ((**D**(**1**)): control group, (**D**(**2**)): As_2_S_2_ group, (**D**(**3**)): co-culture of As_2_S_2_ and inhibitor SB203580, (**D(4**)): co-culture of As_2_S_2_ and inhibitor NAC). $\bar{X}$ ± s; *n* = 3; ** *p* < 0.01 vs. control group; ^▲^ *p* < 0.01 vs. As_2_S_2_ group; ^▲▲^ *p* < 0.01 vs. As_2_S_2_ group.

To assess the effect of As_2_S_2_ on SKM-1 cell apoptosis and cell cycle progression, we selected the IC_50_ (4 μM) at 48 h, which showed a stronger inhibitory effect, as the treatment concentration of As_2_S_2_. SKM-1 cells were treated for 48 h, and apoptosis levels and cell cycle distribution were assessed by flow cytometry. Flow cytometry results showed a significant increase in apoptosis in the As_2_S_2_-treated group compared to the control group (Figure 2A,B), and the percentage of cells in the S-phase was significantly higher (Figure 2C,D). These results suggest that As_2_S_2_ induces apoptosis and promotes S-phase arrest in SKM-1 cells.

### 3.3. Effect of As_2_S_2_ on Apoptosis and Cell Cycle Regulatory Proteins in SKM-1 Cells

**Figure 3:**
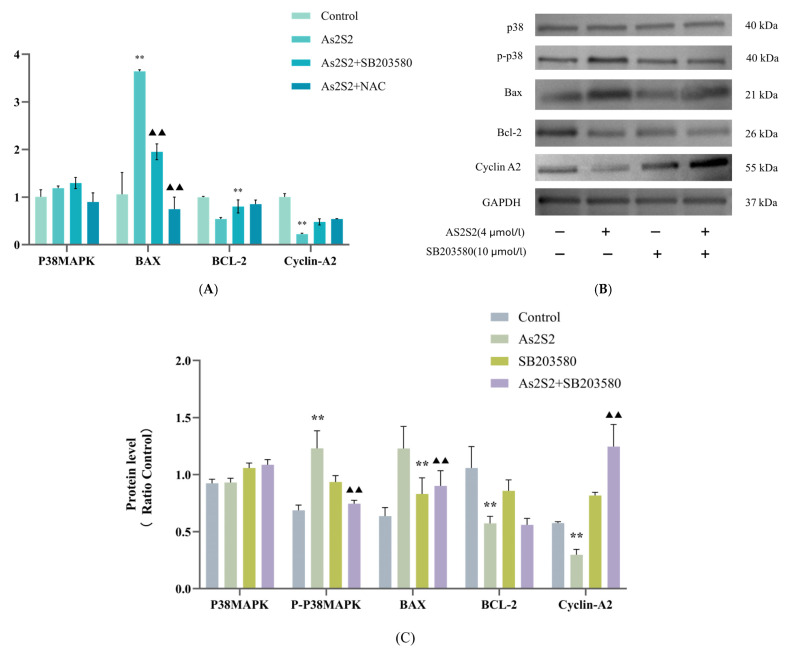
Effect of co-culture of As_2_S_2_ and inhibitors SB203580 (10 μM/L) and NAC (300 mM/L) on apoptosis and cell cycle-regulated proteins in SKM-1 cells. (**A**) Effect of co-culture of the As_2_S_2_ group and inhibitor on the transcript levels of P38 MAPK, BAX, BCL-2, and cyclin A2 mRNA in SKM-1 cells. (**B**,**C**) Effect of co-culture of As_2_S_2_ and inhibitor SB203580 on protein expression levels and quantitative analysis in SKM-1 cells. ($\bar{X}$ ± s; *n* = 3) ** *p* < 0.01 vs. control group; ^▲▲^ *p* < 0.01 vs. As_2_S_2_ group.

To further investigate the molecular mechanisms underlying the effects of As_2_S_2_ on apoptosis and cell cycle progression in SKM-1 cells, we examined the transcriptional and protein expression levels of apoptosis and cell cycle-regulated genes using Western blot (WB) and q-PCR. The q-PCR results showed a significant increase in the transcript levels of the pro-apoptotic protein BAX mRNA and a significant decrease in the transcript levels of the anti-apoptotic protein Bcl-2 and the S-phase regulatory protein cyclin A2 compared to the control group (Figure 3A). The WB results were consistent with the q-PCR data (Figure 3B,C). These findings suggest that As_2_S_2_ induces apoptosis in SKM-1 cells by up-regulating the pro-apoptotic protein BAX and down-regulating the anti-apoptotic protein Bcl-2. Additionally, the ability of As_2_S_2_ to promote S-phase arrest in the cell cycle is associated with the down-regulation of the S-phase regulatory protein cyclin A2.

### 3.4. As_2_S_2_ Promotes ROS Accumulation to Activate the P38 MAPK Signaling Pathway

**Figure 4:**
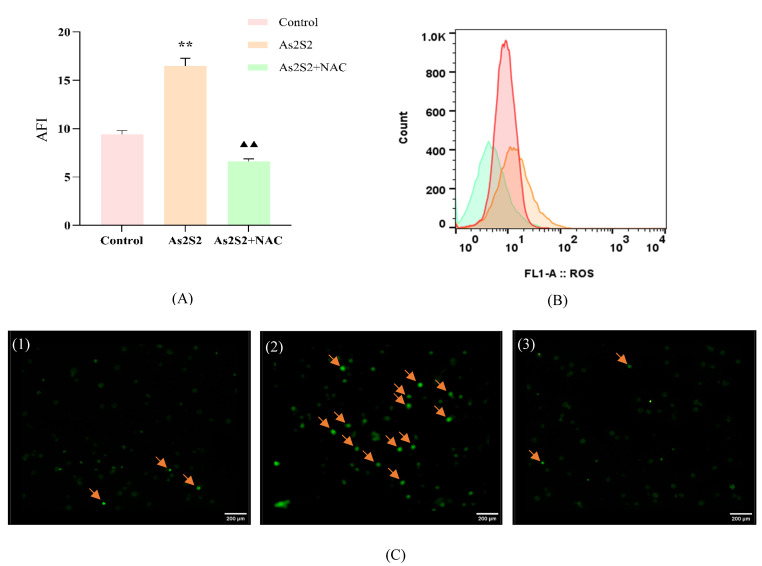

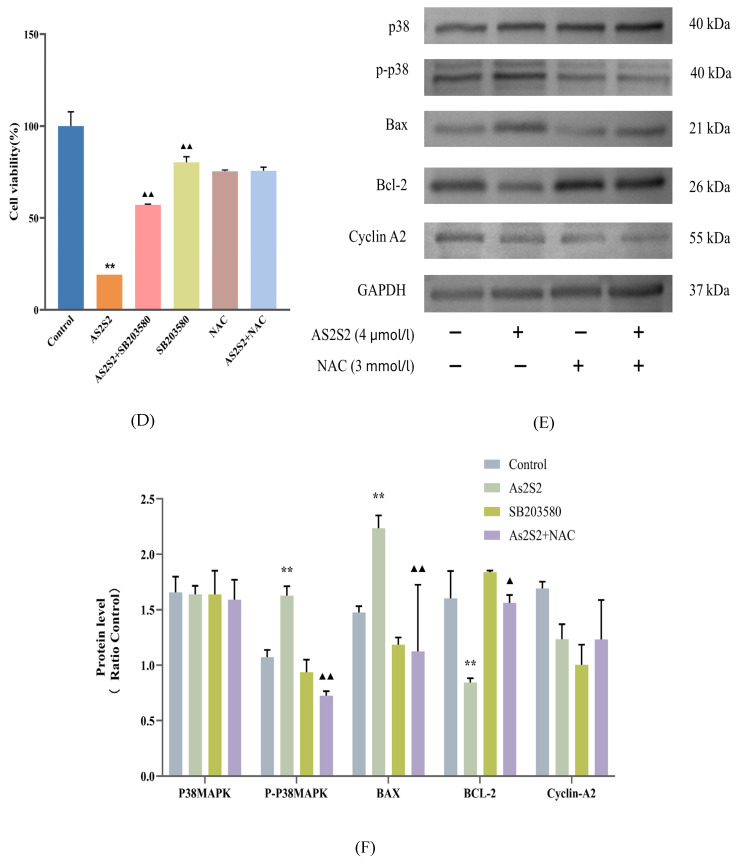
Effect of co-culture of As_2_S_2_ and ROS inhibitor NAC on SKM-1 cells. (**A**) Levels of ROS in SKM-1 cells as measured by flow cytometry. (**B**) Flow fluorescence histogram of ROS. (**C**) The expression level of ROS in SKM-1 cells observed under a fluorescence microscope (100×, (**C**(**1**)): control group, (**C**(**2**)): As_2_S_2_ group, (**C**(**3**)): As_2_S_2_ + NAC group). Arrows indicate cells exhibiting pronounced DCF fluorescence, signifying elevated reactive oxygen species (ROS) levels. (**D**) Effects of co-culture of As_2_S_2_ with SB203580 (P38 inhibitor, 10 μM/L) and NAC (ROS inhibitor, 300 mM/L) on cell viability of SKM-1 cells for 48 h. (**E**,**F**) Effect of co-culture of As_2_S_2_ and ROS inhibitor NAC on protein expression levels and quantitative analysis. ($\bar{X}$ ± s; *n* = 3) ** *p* < 0.01 vs. control group; ^▲^ *p* < 0.01 vs. As_2_S_2_ group; ^▲▲^ *p* < 0.01 vs. As_2_S_2_ group.

Using the IC_50_ (4 μM) at 48 h as the drug dose, cell viability was partially reversed after co-culture with As_2_S_2_ and the P38 MAPK pathway inhibitor (SB203580, 10 μM/L) (Figure 4D). Flow cytometry results showed a significant reduction in the ability of As_2_S_2_ to induce apoptosis and S-phase arrest in the presence of SB203580 (Figure 2A,C). The q-PCR results indicated that the transcript levels of the pro-apoptotic protein BAX mRNA were significantly decreased, while the transcript levels of the anti-apoptotic protein Bcl-2 mRNA were significantly increased compared to the As_2_S_2_ group (Figure 3A). The WB results were consistent with the q-PCR findings and further revealed that the phosphorylation level of P38 MAPK was significantly reduced after the use of the inhibitor (Figure 3B,C). These results suggest that As_2_S_2_ activates the P38 MAPK signaling pathway by promoting the phosphorylation of P38 MAPK, which in turn promotes apoptosis and S-phase arrest in SKM-1 cells, significantly reducing cell viability.

To investigate the relationship between ROS and the P38 MAPK pathway, we examined the effect of As_2_S_2_ on ROS levels in SKM-1 cells using flow cytometry and immunofluorescence. Both methods showed that As_2_S_2_ significantly increased ROS levels in SKM-1 cells compared to the control group (Figure 3A,C). After treating the cells with the ROS scavenger NAC (300 mM/L), both apoptosis levels and S-phase arrest were reversed to a greater extent than in the SB203580 and As_2_S_2_ co-culture group when compared to the As_2_S_2_ group (Figure 2A,C). The q-PCR results showed that compared with the As_2_S_2_ group, NAC-treated cells showed a significant decrease in the level of BAX mRNA transcripts, a significant increase in the level of cell cycle protein A2 and BCL-2 mRNA transcripts, and no significant change in the level of P38 MAPK mRNA transcripts (Figure 3A). Notably, WB results revealed a significant decrease in the phosphorylation levels of P38 MAPK (Figure 4E,F). These findings suggest that the activation of the P38 MAPK pathway by As_2_S_2_ is partially regulated by ROS.

## 4. Discussion

As_2_S_2_ is a major component of the traditional Chinese medicine Andrographis paniculata and has been widely used in Chinese clinical practice for the treatment of hematologic malignancies and solid tumors, such as breast cancer and osteosarcoma, with promising therapeutic effects [17,18]. Although previous studies have demonstrated the antitumor properties of As_2_S_2_ [19], its therapeutic mechanism in MDS remains to be fully elucidated.

To investigate the potential mechanism of As_2_S_2_ on SKM-1 cells, we analyzed SKM-1 cells for apoptosis and cell cycle correlation after treatment with As_2_S_2_ at a concentration of 4 μM (close to the IC_50_ concentration at 48 h) for 48 h. The results demonstrated that As_2_S_2_ reduced the viability of SKM-1 cells in a time- and dose-dependent manner and activated the P38 MAPK signaling pathway by promoting ROS accumulation, which subsequently induced apoptosis and S-phase arrest in MDS cells.

Abnormal regulation of the cell cycle is a hallmark of cancer. Physical and chemical factors, such as environmental stress, radiation, and drugs, can cause cellular DNA damage leading to genomic instability and cell cycle disruption [20]. Cell cycle checkpoints function as DNA surveillance mechanisms during cell division, effectively preventing the accumulation of erroneous genetic information [21]. Therefore, blocking the cell cycle and inducing cell cycle arrest could be an effective strategy for cancer treatment [22]. Our results demonstrated that As_2_S_2_ induces S-phase arrest in SKM-1 cells by down-regulating cyclin A2 protein. Previous studies have shown that cyclin A2 maintains DNA replication by binding to CDK2 to form the CDK2-cyclin A2 complex, which facilitates the S/G2 phase transition and promotes tumor cell proliferation [23]. Furthermore, treatment of colon cancer cells (Caco-2) with the CDK2 inhibitor Roscovitine significantly reduced Caco-2 cell proliferation and survival [24], which is consistent with our findings.

Cell cycle arrest is one of the key triggers of apoptosis [25]. Apoptosis is a form of programmed cell death regulated by multiple signaling pathways [26], with the death receptor-mediated extrinsic pathway and the mitochondria-mediated intrinsic pathway being the two primary mechanisms of apoptosis regulation [27,28]. ROS influence both apoptotic pathways; they can induce apoptosis via the extrinsic pathway by activating cell-surface death receptors or inducing endoplasmic reticulum stress [29], and through the intrinsic pathway by causing mitochondrial damage, promoting apoptotic vesicle formation, and inducing DNA damage, ultimately leading to apoptosis [30]. In our study, As_2_S_2_-treated cells exhibited elevated ROS levels, accompanied by increased apoptosis and S-phase arrest. To further investigate the role of ROS, we co-cultured the ROS scavenger N-acetylcysteine (NAC) with As_2_S_2_ and measured ROS levels in SKM-1 cells. The results showed that ROS levels were reduced, and both apoptosis and S-phase arrest were partially reversed, suggesting that ROS accumulation induced by As_2_S_2_ is a key contributor to apoptosis and cell cycle alterations.

A complex interaction exists between reactive oxygen species (ROS) and the mitogen-activated protein kinase (MAPK) signaling pathway. The MAPK signaling pathway is activated upon excessive reactive oxygen species (ROS) accumulation triggered by environmental stressors, pharmacological induction, or other stimulatory factors [31]. Previous studies have shown that the proliferation, differentiation, and apoptosis of tumor cells are closely linked to the phosphorylation and regulation of protein kinases within the MAPK signaling pathway, influencing the progression of hematological malignancies [32]. P38 MAPK is a key branch of the MAPK signaling pathway that is continuously activated and phosphorylated in response to oxidative stress and inflammatory stimuli. It promotes apoptosis by mediating the upregulation of Bax and the downregulation of Bcl-2 through the activation of caspase 9 and caspase 3, thereby triggering downstream apoptotic signaling [33]. In this study, SKM-1 cells treated with As_2_S_2_ exhibited increased phosphorylation of P38 MAPK, upregulation of the pro-apoptotic protein BAX, and downregulation of Bcl-2, leading to enhanced apoptosis: as confirmed by flow cytometry results. Although we used the IC_50_ (4 μM) at 48 h as the intervention condition, the apoptosis rate did not reach the expected rate of about 50% (actually about 30%). This discrepancy could be attributed to As_2_S_2_-induced cell cycle arrest or the potential involvement of alternative cell death mechanisms (e.g., ferroptosis or pyroptosis) in a subset of cells, which warrants further investigation.

To further investigate whether As_2_S_2_ exerts its effects through the ROS/P38 MAPK signaling pathway, we treated SKM-1 cells with As_2_S_2_ and analyzed the phosphorylation level of P38 MAPK as well as the expression levels of BAX, BCL-2, and cyclin A2. The results demonstrated that As_2_S_2_ treatment alone led to increased apoptosis and S-phase arrest, along with the significant upregulation of phosphorylated P38 MAPK, increased BAX expression, and decreased BCL-2 and cyclin A2 expression. However, when co-cultured with inhibitors, the effects of As_2_S_2_ on apoptosis and phosphorylated P38 MAPK levels were partially reversed, along with corresponding changes in BAX, BCL-2, and cyclin A2 protein expression levels.

A limitation of this study is the use of a single cell line, which may limit the generalizability of our findings. Using only the SKM-1 cell line fails to fully capture the complexity and diversity of cellular behaviors and responses across different biological environments. Future studies should incorporate more cell lines from diverse tissue sources or represent different stages of MDS pathogenesis to validate and extend the current results. This would enhance the robustness of the conclusions and provide a more comprehensive understanding of the underlying mechanisms.

In conclusion, we hypothesize that As_2_S_2_ promotes ROS accumulation and activates the ROS/P38 MAPK signaling pathway through the phosphorylation of P38 MAPK, thereby enhancing apoptosis and inducing S-phase arrest in the cell cycle. This mechanism suggests that As_2_S_2_ may serve as a potential therapeutic agent for MDS and that the ROS/P38 MAPK pathway could be a promising target for MDS treatment.

## Figures and Tables

**Table 1:** Primer sequences for target genes.

Primer	Sequence 5’ → 3’
P38 MAPK	TGCGTCTGACAGGAACACCTCTTCTCCAGCAAGTCGACAG
BAX	GCCCTTTTGCTTCAGGGTTTCCACTCGCTCAGCTTCTTGGT
BCL-2	TGTCCCTTTGACCTTGTTTCTTCATTTGCCATCTGGATTTT
Cyclin-A2	CTGCTCCAACAGTAAATCAGTTTCAAGGCAGCTCCAGCATAAC

## Data Availability

The SKM-1 cell line was generously provided by the First Affiliated Hospital of Soochow University.
